# Toughening Enhancement Mechanism and Performance Optimization of Castor-Oil-Based Polyurethane Cross-Linked Modified Polybutylene Adipate/Terephthalate Composites

**DOI:** 10.3390/ma16186256

**Published:** 2023-09-18

**Authors:** Qing Zhang, Jin Huang, Na Zhou

**Affiliations:** 1 State Key Laboratory Incubation Base for Green Processing of Chemical Engineering, School of Chemistry and Chemical Engineering, Shihezi University, Shihezi 832003, China; 2 School of Chemistry and Chemical Engineering, Chongqing Key Laboratory of Soft-Matter Material Chemistry, Southwest University, No. 2 Tiansheng Road, Beibei, Chongqing 400715, China

**Keywords:** melt blending, castor oil, TDI, composite modification, PBAT-based complex

## Abstract

In this study, polyol castor oil (CO) and toluene-2,4-diisocyanate (TDI) were selected to modify PBAT, and castor-oil-based polyurethane (COP) was produced in a PBAT matrix using melt-blending and hot-pressing technology to study the effect of network cross-linking structure on various properties of bio-based polyester PBAT, aiming to introduce CO and TDI to improve the mechanical properties of composite materials. The results showed that when the total addition of CO and TDI was 15%, and the ratio of the hydroxyl group of CO to the isocyanate group of TDI was 1:1, the mechanical properties were the best. The tensile strength of the composite was 86.19% higher than that of pure PBAT, the elongation at break was 70.09% higher than that of PBAT, and the glass transition temperature was 7.82 °C higher than that of pure PBAT. Therefore, the composite modification of PBAT by CO and TDI can effectively improve the heat resistance and mechanical properties of PBAT-based composites.

## 1. Introduction

In recent years, with the rapid development of the global economy and technology, the plastics industry has played an important role. However, over time, the production and use of plastic products have had a serious impact on the environment [1]. These plastic products are difficult to degrade, and the cost of disposal is high, so most of them are incinerated or landfilled, which not only causes a lot of environmental pollution and economic losses [2,3], but the incineration of polymers also releases polluting gases, and the released carbon dioxide aggravates the greenhouse effect, resulting in secondary pollution [4]. At the same time, the way of landfill destroys the surrounding ecological environment system [4,5] and waste land resources, to a certain extent. Therefore, in order to solve the problem of plastic pollution and pursue a more sustainable future, biodegradable plastics are widely promoted and used [6,7].

Biodegradable plastic is a special kind of plastic; it has good performance and can be decomposed naturally after being discarded, which means that it adds no pollution to the environment [8,9,10]. At present, there are various biodegradable plastics, for example, polylactic acid (PLA), polyhydroxy alkanoates (PHA), poly-β-hydroxybutyrate (PHB), polycaprolactone (PCL), polybutylene adipate/terephthalate (PBAT), etc. PBAT is a typical aromatic–aliphatic biodegradable polyester, which is synthesized from butylene glycol, adipic acid and terephthalic acid. A degradable plastic that can be decomposed into carbon dioxide, water and biomass by microorganisms in the natural environment without causing pollution in the environment. However, the price of PBAT is about twice, or even three times, that of traditional plastics such as polyethylene [11], and the mechanical properties are not as good as some traditional plastics [12]. Therefore, the PBAT market will only be more promising and advantageous when production costs are reduced and performance is improved [12,13].

At present, many studies have been carried out on the compound modification of PBAT. Song [14] et al. added styrene–acrylonitrile–glycidyl methacrylate terpolymer (SAG) as a chain extender to PBAT and used it for foaming. It was found by infrared spectroscopy that the chain-extended modified component had a characteristic peak of the C-H bond outside the benzene ring at 698 cm^−1^. Gel permeation chromatography showed that with the increase in SAG content, PBAT began to show a micro-cross-linked phenomenon, and the gel content reached 0.47 at the SAG content of 5 wt%. Tang [15] et al. used the multifunctional epoxy-type chain extender (Joncyl ADR 4370), which was added to PBAT and blown into a mulch film. The introduction of ADR formed a cross-linked network structure in PBAT, and these network structures can slow down the film. Aging in the environment can also slow down the decline in tensile strength caused by aging, allowing the performance to be maintained for a longer period of time. Cui [16] et al. used EGMA as a crystalline chain extender to modify PBAT, and the results showed that the introduction of this chain extender made the PBAT foam material have a bimodal foam structure because the crystal and amorphous interface of PBAT was produced, and homogeneous and heterogeneous nucleation sites were identified. Homogeneous nucleation sites could induce the formation of large cells, and heterogeneous nucleation sites could induce the formation of small cells. Wang [17] et al. melt blended PBAT and poly(glycolic acid) PGA in a screw and injected the sample through an injection molding machine and used 4-4’-methylene diphenyl diisocyanate (MDI) as a compatibilizer to study the performance of the composite. The notched impact strength increased significantly from 9.0 kJ m^−2^ to 22.2 kJ m^−2^ when the PBAT content reached 60 wt%, and as the PBAT content increased to 70 wt%, the notched impact strength reached the highest, which was 56.6 kJ·m^−2^. Nayak [18] prepared PBAT using fusion layer intercalation technology and added organically modified nanoclay and thermoplastic starch (TPS) to PBAT to prepare composite materials. Compared with pure PBAT, adding 30% of TPS and nanoclay, the post-clay tensile modulus and elongation at break increased by 44.45% and 776.9%, respectively. With the increase in nanoclay, the storage modulus and glass transition temperature of PBAT increased, which also confirmed the higher biodegradability of PBAT in TPS and nanoclay.

PBAT research has shown a linear growth trend since it entered people’s field of vision, usually showing low strength, low toughness and a relatively high price, resulting in extremely limited applications [19,20,21]. The application fields of PBAT [22] mainly include the packaging industry: fresh food packaging, express bags, shopping bags, garbage bags, etc.; the agricultural field: agricultural mulching, planting bags, pesticide packaging, etc.; the medical field [23]: disposable medical equipment, surgical supplies, medical packaging, etc.; and the automobile industry: automotive interior parts, tires, parts, etc. Castor oil is an abundant renewable resource [24]. As a polyol, it has multifunctional groups and a flexible chain structure. Theoretically, it can be synthesized into bio-based polyester using melt condensation [25,26]. Polymer materials directly prepared from castor oil are generally highly cross-linked and have a flexible network structure. Informed by relevant research work and reports [27,28,29], during the melt-blending process, the cross-linking of castor oil and TDI can occur rapidly, and a tough blend can be formed, with the castor-oil-based polyurethane as the dispersed phase and the polyester matrix as the continuous phase. In addition, due to the high fluidity of castor oil during the melt-blending process, it is easily leaks during processing, which affects the final performance of the composite material; therefore, we need to use TDI and castor oil for rapid cross-linking in order to reduce the waste of castor oil [30]. Due to the presence of reactive NCO groups, diisocyanates are widely used as reactive agents. Therefore, the choice of TDI is the optimal way to improve the compatibility of the two-phase interface [31,32,33,34,35]. We introduced castor oil and TDI into the PBAT matrix, and the two reacted in situ in PBAT to form a castor-oil-based polyurethane cross-linked network structure, and we then explored the influence of PBAT on the mechanical properties, thermal performance, crystallization properties, rheological properties, etc., and the mechanism of strengthening and toughening was analyzed at a microscopic level.

## 2. Experimental

### 2.1. Materials

The PBAT model used in this study is ECO-A05, the specification is 1.22 g cm^−3^, and the manufacturer is China Dongguan Changchong Plastics Co. TDI, analytically pure; castor oil, AR 83–88%, molecular weight: 933.43, CAS: 8001-79-4. The experimental apparatus is shown in Table 1.

### 2.2. Sample Preparation

#### 2.2.1. Preparation of PBAT-CO/TDI Composite

PBAT is vacuum-dried at 80 °C for 8 h to achieve the purpose of water removal. Calculate and weigh castor oil (CO) and TDI according to the ratio of -OH in castor oil and -NCO in TDI 1:1, and the mass fractions of fillers in PBAT matrix are 5%, 10%, 15%, 20%, 25%, 30%. The specific processing parameters of the internal mixer are as follows: the rotor speed of the internal mixer is 80 rpm, and the internal mixing temperature is 150 °C. The specific operation is first heat and melt the dried PBAT in the internal mixer, then add the corresponding proportion of CO and banbury together for 5 min, then add the corresponding proportion of TDI and continue blending in the internal mixer for 15 min, then take out and banbury. The mixed material is cooled to room temperature, cut into small pieces with scissors and further pulverized into small particles with a high-speed universal pulverizer. Before hot pressing, the high-speed pulverized particles are dried in an oven at 80 °C for 4 h. The pulverized particles are paved on the mold by hand, pressurized to 5 MPa, and the pressure is released 3 times to eliminate air bubbles and then heated at a temperature of 150 °C for 6 min, then cold pressed to room temperature to release the pressure, demolded to obtain dumbbell-shaped splines, and the composite samples are marked as PBAT-CO/TDI-5, PBAT-CO/TDI-10, PBAT-CO/TDI-15, PBAT-CO/TDI-20, PBAT-CO/TDI-25, PBAT-CO/TDI-30.

#### 2.2.2. Preparation of Gel (COP)

Weigh about 2 g of the complex sample and soak it in chloroform for 1 week. The dissolved part is precipitated with excess methanol reagent, and a white flocculent precipitate is precipitated, and the white powder floc is fully dried in an oven, and the insoluble part is separated by filtration and then it is rinsed with chloroform reagent 3–5 times and fully dried at 80 °C for 12 h [30] to obtain the castor-oil-based polyurethane gel (COP) part of the complex.

### 2.3. Performance Testing and Characterization

Infrared spectrum test uses RF-5301PC (Shimadzu, Japan) infrared spectrometer, the scanning wavelength range is 400–4000 cm^−1^, and the number of scanning is 32 times.

The differential scanning calorimetry test is carried out with a DSC-214analyzer, and the specific operation is as follows: weigh about 7.0 mg of the sample, place it in an aluminum crucible, and heat it at 10 °C/min to raise the temperature from 30 °C to 200 °C and maintain for 3 min; then, decrease to −50 °C at 10 °C/min and maintain for 3 min to obtain a cooling curve; finally, raise to 200 °C at 10 °C/min to obtain a second heating curve.

X-ray diffraction test (XRD) is tested with a D8-ADVANCE diffractometer with a voltage of 36 kV and a current of 20 mA, and the scanning range is set between 5° and 80°. The step size of each scan is 0.01°, and the scan speed is 4°/min.

The thermogravimetric test is performed with a Q500 thermogravimetric analyzer. First, a sample of about 7.0 mg is weighed, and the thermal stability of the PBAT-ABF/TDI composite is tested using a thermogravimetric analyzer. Under a nitrogen atmosphere, the temperature is raised from 30 °C to 800 °C at a rate of 10 °C/min.

A rotational rheometer (TA DHR-1) is used to test the rheological properties of PBAT and the composite PBAT-CO/TDI. The oscillation mode is used, the temperature is set at 150 °C, and the frequency scanning range is set at 0.01–100 Hz/s. The strain is 0.5%.

The dynamic thermomechanical properties of the composite PBAT-CO/TDI are tested and analyzed using a DMA tester (Q800). During the test, use a special cutter to cut the sample to be tested into a sample with a length of 12 mm and a width of 1 mm. The parameter settings during the measurement are as follows: the temperature setting range is from −60 °C to 100 °C, the heating rate is 3 °C/min, the stretching frequency is 1 Hz, the fixed stretching range is 0.1%, the static force value is 0.01 N, and the strain tracking is set to 125%. The curves of storage modulus, loss modulus and loss factor with temperature are obtained, respectively.

Test the tensile properties of the composite PBAT-CO/TDI on a universal testing machine (Sansi CMT6503). The tensile speed of the standard sample is set to 10 mm/min, and the ambient temperature is at about 30 °C [36]. Measure the data of 5 standard dumbbell-shaped splines and, finally, take the average value as the final data.

The microstructure of the samples is observed using a S4800 scanning electron microscope. The hot-pressed standard specimens are placed at room temperature for 4 h, then soaked in liquid nitrogen for 1 h, and then quenched quickly to preserve the complete section, which is sprayed with gold.

## 3. Results and Discussion

### 3.1. Mechanical Properties of Composite Materials

**Figure 1:**
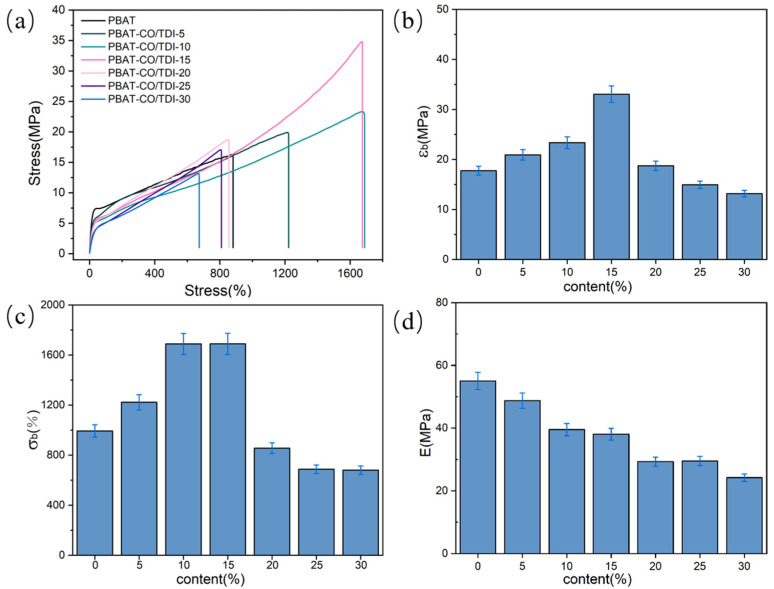
PBAT and PBAT-CO/TDI composites: (**a**) stress–strain curve, (**b**) tensile strength, (**c**) elongation at break and (**d**) modulus.

Figure 1 shows the mechanical properties of pure PBAT and composite PBAT-CO/TDI. As shown in the figure, the elongation at break and tensile strength of pure PBAT are 17.75 MPa and 993.18%, respectively, and the tensile modulus is 55.04 MPa. With the increase in CO/TDI filler content, the mechanical properties of the composite enhance. When the content of CO/TDI filler is 15%, the elongation at break and tensile strength of the composite are optimal, which are 1689.36% and 33.05 MPa, respectively. Compared with pure PBAT, the tensile strength is increased by 86.19%, the elongation at break increased by 70.09%, and when the content of CO/TDI filler was 5%, the tensile strength of the composite was 20.92 MPa, which was 17.85% higher than that of pure PBAT, and the elongation at break was 1222.68%, which was 23.10% higher than that of pure PBAT. When the content of CO/TDI was 10%, the tensile strength of the composite was 23.36 MPa, and the elongation at break was 1689.05%, which were 31.60% and 70.06% higher than that of pure PBAT, respectively. As the castor-oil-based polyurethane (COP) generated by cross-linking in the PBAT matrix can increase the force between the polymer molecular chains, it can enhance the interaction between the PBAT molecular chains and can also prevent the slipping and breaking of the PBAT molecular chains. The phase interface force is improved, and the PBAT crystal structure is denser and orderly, and the mechanical properties of the composite are improved. However, when the filling content is 20–30%, the cross-linking structure of the polyurethane cross-linking network is excessive, and the tensile strength of PBAT will decrease because the cross-linking density will increase the number of cross-linking points in the matrix, which can lead to a decrease in the overall strength of the material. Excessive cross-linking may cause the connection between cross-linking points to become chaotic, which makes it difficult for the material to disperse the stress evenly when it is stressed, resulting in a decrease in strength. Excessive cross-linking also interferes with the crystallization behavior of polymer chains, resulting in a decrease in crystallinity, which affects the tensile strength of the material. In addition, excessive cross-linking can also lead to microscopic defects in the material, which can act as stress concentrators when the material is stressed, making the material more prone to cracking and thus reducing tensile strength. Therefore, when controlling the polyurethane cross-linking network in the PBAT matrix, it is necessary to balance the relationship between the cross-linking density and the overall properties of the material to ensure that the resulting material has the desired properties.

### 3.2. Structural Characterization of Composite Materials

**Figure 2:**
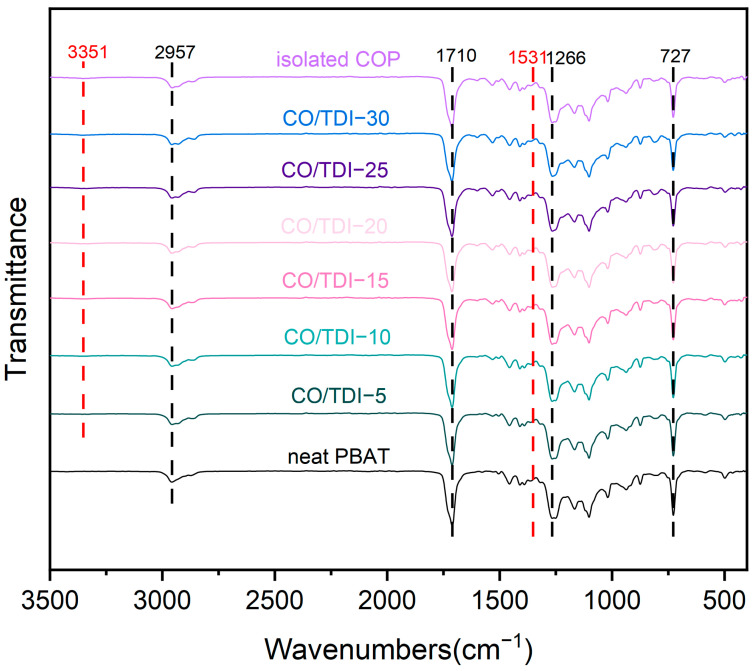
Infrared spectra of PBAT and PBAT−CO/TDI composite.

Figure 2 shows the FTIR spectra of pure PBAT, the complex PBAT-CO/TDI and the gel fraction (COP). As shown in the figure, the wavenumbers produce strong stretching vibration peaks at 1710 cm^−1^ and 1266 cm^−1^, which belong to the carbonyl (C=O) and ester group (C-O) of PBAT, and the absorption peak at 727 cm^−1^. The out-of-plane bending vibration absorption peak corresponding to C-H on the para-disubstituted benzene ring indicates the existence of the benzene ring. The absorption peak at 1456 cm^−1^ corresponds to the moderate-intensity bending vibration peak of -CH_2_, and the absorption peak at 2957 cm^−1^ belongs to the stretching vibration peak of methylene. As the hydroxyl content in PBAT is very small and only exists at the end of the molecular chain, there is almost no absorption peak of -OH in the infrared curve of pure PBAT.

**Figure 3:**
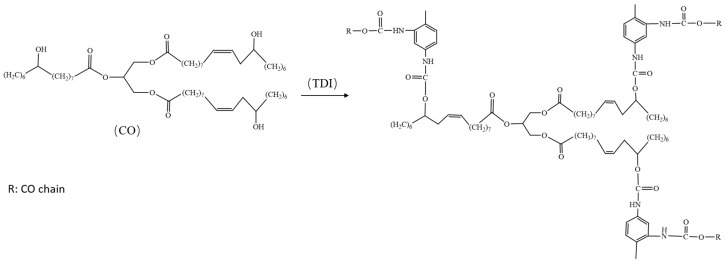
Reaction mechanism of castor oil and TDI.

The characteristic absorption peaks of the infrared spectrum of the gel part at 3351 cm^−1^ and 1531 cm^−1^ are amide bonds and N-H bonds. These two characteristic absorption peaks can also be found in all composite samples but not in pure PBAT, indicating that COP was successfully formed in the PBAT matrix during the melt-blending process. No characteristic absorption peak of the isocyanate group at 2200 cm^−1^ was found in all composite samples and gel samples, indicating that TDI can be completely reacted during the melt-blending process, during the melt blending process castor oil reacts in situ with TDI to form a cross-linked COP as shown in Figure 3, and the ester carbonyl in PBAT and the benzene ring coexist in a Yoke effect, so a certain degree of red shift occurs.

### 3.3. Thermal Property (DSC) Analysis of Composite Materials

**Figure 4:**
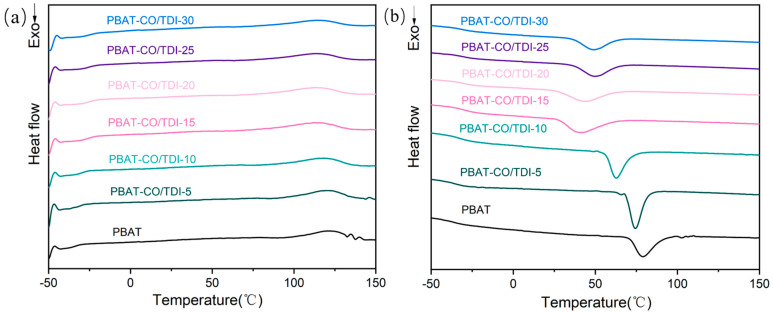
Cooling curves (**a**) and quadratic heating curves (**b**) of PBAT and PBAT-CO/TDI composites.

Figure 4 is the DSC cooling curve and secondary heating curve of pure PBAT and composite PBAT-CO/TDI, and Table 2 shows the specific data of the DSC test of pure PBAT and composite PBAT-CO/TDI. The glass transition temperature of the composite can also reflect the degree of compatibility between different components and the strength of the interfacial force. As shown in the table, the glass transition temperature of pure PBAT is −36.4 °C, and for the composite PBAT-CO/TDI, with the increase in CO/TDI filler content, the glass transition temperature first increases and then decreases, but all are higher than pure PBAT. The glass transition temperature of PBAT-CO/TDI-15 reaches the highest at −26.0 °C, which is 10.4 °C higher than that of pure PBAT, and the glass transition temperature of PBAT is the largest. The greater the difference between the glass transition temperature of the composite and pure PBAT, the better the two-phase compatibility of the composite; therefore, the two-phase compatibility of the composite PBAT-CO/TDI-15 and pure PBAT is the highest.

In addition, the cold crystallization temperature of all composite samples is lower than that of pure PBAT and decreases gradually with the increase in CO/TDI filler content. A decrease in the cold crystallization temperature represents a faster crystallization rate, which indicates that the crystallization rate increases and the crystallinity increases after PBAT is modified. The crystallinity of pure PBAT is 7.5%, and the crystallinity of composite PBAT-CO/TDI-15 is the largest, which is 14.6%. χ_c_DSC (%) increased first and then decreased, which is consistent with the change rule of Tc (°C). It shows that the COP generated in the complex PBAT-CO/TDI promotes the crystallization of PBAT because the generated COP plays the role of heterogeneous nucleation, which can nucleate the crystallization of PBAT at the two-phase interface.

### 3.4. X-ray Diffraction (XRD) Analysis of Composite Materials

**Figure 5:**
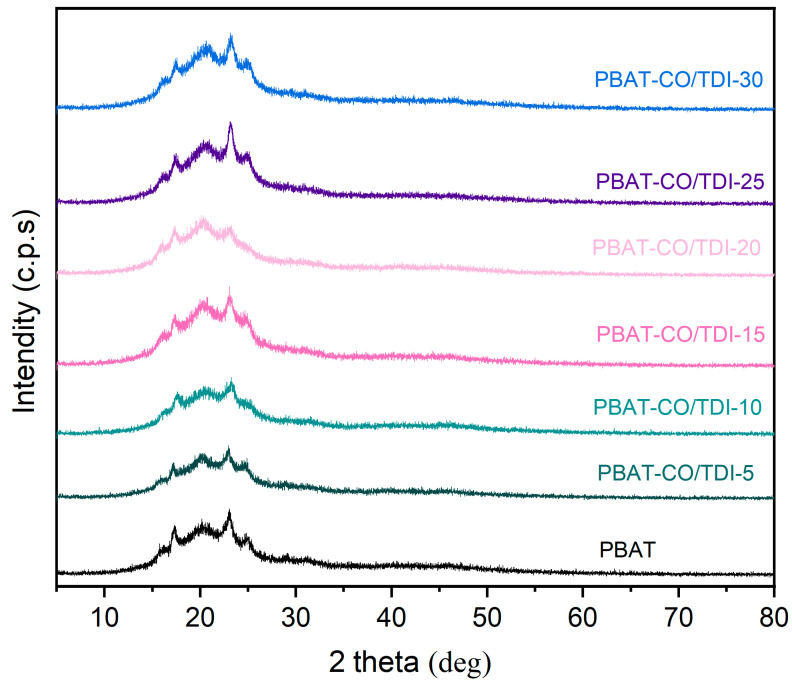
XRD patterns of PBAT and PBAT-CO/TDI complex.

The properties of polymer materials are often closely related to their crystal structures. Therefore, X-ray diffraction analysis can provide an in-depth understanding of the structural properties of polymer materials and provide a basis for further optimizing the properties of polymer materials. As shown in Figure 5, the five characteristic diffraction peaks of PBAT are at 2θ of 16.27°, 17.33°, 20.15°, 23.06° and 24.83°. The figure shows that the XRD diffraction curves of pure PBAT and composite materials are closely similar, and there is no significant difference. The addition of CO/TDI filler does not make the material show new diffraction peaks or the disappearance of diffraction peaks; however, the intensity of the diffraction peaks change a little bit, which indicates that the addition of fillers has no effect on the crystal morphology and crystal phase but that the appropriate amount of fillers promotes the growth of PBAT crystals. 

### 3.5. Thermogravimetric (TG) Analysis of Composite Materials

**Figure 6:**
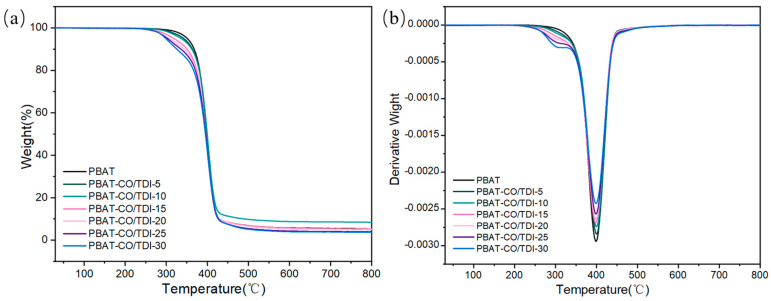
PBAT and PBAT-CO/TDI composites: (**a**) TG curve, (**b**) DTG curve.

Figure 6 presents the TG and DTG diagrams of PBAT and the complex PBAT-CO/TDI. As shown in the figure, the COP produced by CO and TDI in the PBAT matrix has good fluidity, which can promote the decomposition of PBAT when it is thermally decomposed, so that the carbon in the decomposition products of PBAT can be taken away more easily. The two phases of the polyurethane cross-linked network and PBAT have good compatibility, and the two can form interpenetrating phases to promote the thermal decomposition of PBAT. Therefore, the addition of COP can reduce the carbon residue rate during the thermal decomposition of PBAT and improve the degradability and environmental performance of PBAT-based composites.

Table 3 shows the thermogravimetric specific data of PBAT and composite PBAT-CO/TDI. With the increase in CO/TDI filler content, the initial degradation temperature of the polymer decreases, and the initial degradation temperature of the composite PBAT-CO/TDI-30 is the lowest, 298.66 °C, which is 52.64 °C lower than that of PBAT, and the thermal stability decreases. The maximum decomposition temperature of all composites is almost constantly independent of the filler content, which is almost the same as that of PBAT, about 400 °C. This is mainly due to the fact that the maximum decomposition temperature of PBAT is mainly determined by its molecular structure and the volatility of degradation products, while the cross-linked network of castor-oil-based polyurethane has no obvious influence on these factors. The residual carbon rate of PBAT at 600 °C is 5.81%. The carbon residual rate of all composites is lower than that of pure PBAT, and it gradually decreases with the increase in CO/TDI filler content. When the CO/TDI content is 30%, the minimum is 3.96%, indicating that the degradability of the complex has been improved.

### 3.6. Analysis of Rheological Properties of Composite Materials

**Figure 7:**
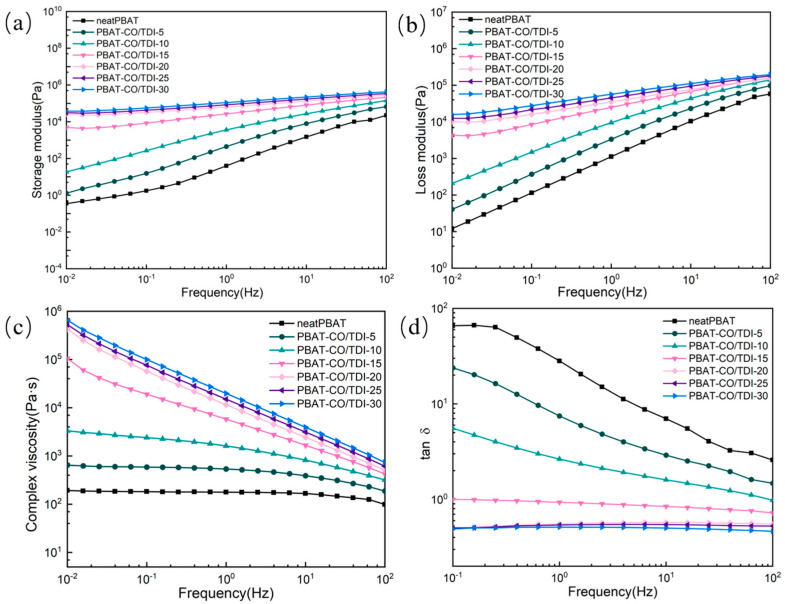
PBAT and PBAT-CO/TDI composites: (**a**) storage modulus, (**b**) loss modulus, (**c**) complex viscosity, (**d**) loss angle tangent.

Figure 7 shows the rheological curves of pure PBAT and complex PBAT-CO/TDI. As shown in the figure, the complex viscosity of the compound increases with the filler content in the low-frequency region, and it belongs to Newtonian fluid characteristics in the whole frequency range for pure PBAT. For the low-filling compound PBAT-CO/TDI, the characteristics of Newtonian fluid in the low-frequency region are very obvious, and the complex viscosity in the high-frequency region shows a slight downward trend. For the compound with a high-filling content, the complex viscosity has no Newtonian fluid parallel region in the whole frequency range, and the higher the frequency, the complex viscosity also decreases linearly, indicating that the rheology of the compound sample with the high-filling content is nonlinear in the whole frequency range, which is because the reaction of CO and TDI in the PBAT matrix leads to the formation of castor-oil-based cross-linked polyurethane during the blending process.

In addition, as the frequency increases, the storage modulus and loss modulus of the composite increase gradually, and both of which are higher than those of pure PBAT. In rheology, the storage modulus is a parameter describing the elastic deformation ability of a material. The larger the storage modulus, the stronger the material’s ability to resist deformation and display better elasticity. In the rheological curve, the loss modulus refers to the energy dissipation degree of the substance under the action of periodic stress, and the loss modulus is an inherent property of the material itself, which is related to the viscoelasticity of the material. Loss modulus is a physical characteristic of a material, which refers to the energy loss absorbed by the material during the deformation process when the material undergoes dynamic deformation. For the composite PBAT-CO/TDI, when the loss modulus increases gradually, the movement ability of the molecular chain decreases, which is because the cross-linked polyurethane (COP) formed in the composite has a network structure and has a great influence on the movement of the matrix molecular chain hindrance. With the increase in COP content, the binding effect of the molecular chain movement of the PBAT matrix in the complex is gradually enhanced, which leads to the gradual increase in the viscosity of the complex [37]. The increase in COP is equivalent to increasing the cross-linking density of the material, which makes the molecular chain movement of the material more constrained, resulting in an increase in viscosity. When the content of CO/TDI filler in the composite is 20%, 25% and 30%, the storage modulus is greater than the loss modulus in the whole frequency range, and a typical solid-like behavior is exhibited. Some researchers have reported that the change in loss tangent with angular frequency can reflect the existence of the gel point of the composite [38,39,40], and the threshold permeability of the polymer composite can be obtained [41,42]. It can be seen from Figure 7d that with the increase in frequency, the loss tangent of PBAT and all composite samples gradually decreases, and the loss tangents of the composites PBAT-CO/TDI-20, PBAT-CO/TDI-25, PBAT-CO/TDI-30 are all ≤1, indicating that the cross-linked network constrains the migration of PBAT molecular chains.

### 3.7. Analysis of Dynamic Thermomechanical Properties of Composite Materials

**Figure 8:**
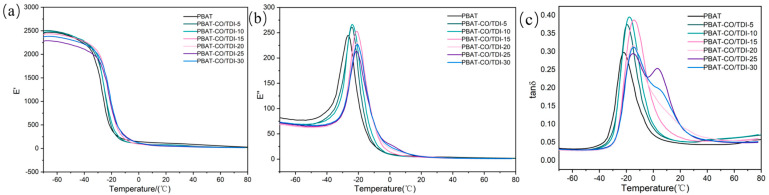
PBAT and PBAT-CO/TDI composites: (**a**) storage modulus, (**b**) loss modulus, (**c**) loss factor.

Figure 8 shows the dynamic thermomechanical analysis diagrams of PBAT and the composite PBAT-CO/TDI, and Table 4 presents the DMA specific data of PBAT and the composite PBAT-CO/TDI. Dynamic thermodynamic analysis is used to study the relationship between the storage modulus, loss modulus, loss factor and temperature of polymer materials under the vibration of tensile mode and under the condition of program temperature control, which can be used to analyze the relationship between polymer materials and performance. As shown in the table, the glass transition temperature of the composite PBAT-CO/TDI increases first and then decreases with the increase in the filler content and reaches the highest at −14.22 °C when the content is 15%, which is 7.82 °C higher than that of pure PBAT. It shows that the composite PBAT-CO/TDI-15 has the best two-phase interface compatibility and the strongest interfacial force. At this time, the heat resistance and degradability of the composite are better than pure PBAT. As shown in Figure 7, the glass transition temperature of all the composites is higher than that of pure PBAT, indicating that the castor-oil-based polyurethane generated by CO and TDI in the PBAT matrix effectively improves the heat resistance of the polymer, and the increase in Tg leads to a gap between the two phases. The interfacial force is enhanced, and at the same time, the free movement of the PBAT polymer molecular chain is restrained.

### 3.8. Microscopic Morphological Analysis of Composite Materials

**Figure 9:**
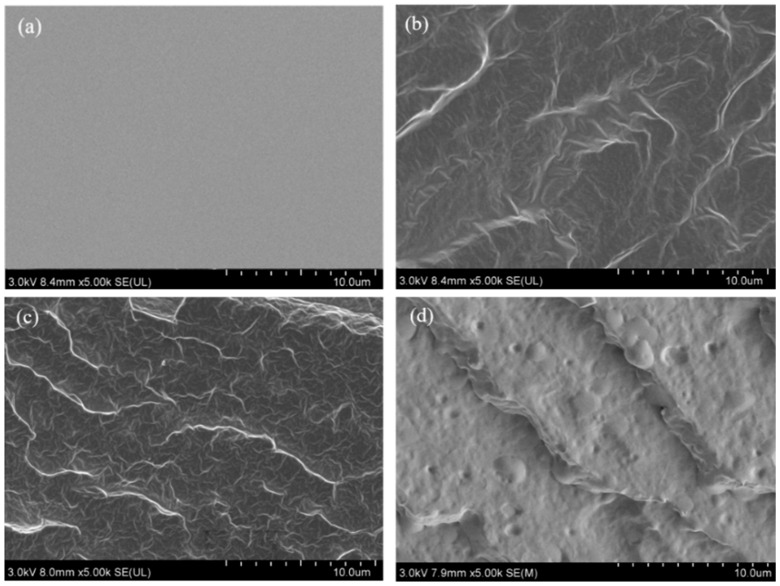
Fracture surface morphologies of (**a**) PBAT, (**b**) PBAT-CO/TDI-5, (**c**) PBAT-CO/TDI-15, (**d**) PBAT-CO/TDI-30.

As shown in Figure 9, the surface of pure PBAT is clean and free of defects after quenching under liquid nitrogen conditions. The composite PBAT-CO/TDI-5 has a microphase separation structure in the scanning image, and the composite PBAT-CO/TDI-15 has a more obvious microphase separation structure, and the microstructure is dense and uniform. From Figure 9c, it can be seen that the CO phase and the PBAT phase are perfectly co-continuous without obvious gaps and become more continuous and dense. The results showed that TDI further reacted with CO to make the PBAT matrix tightly connected, thereby the interfacial adhesion between CO and PBAT is improved, and under the action of TDI, a more continuous and dense cross-linked structure is formed in the PBAT matrix. At the same time, the interface area between the phases can prevent the crack from expanding, thereby improving the elongation at break of the material. The phase interface in the microphase separation structure can reduce the concentration of stress and absorb the energy generated during the crack propagation, thereby slowing down the crack. This energy absorption mechanism can convert the shear stress into the elastic deformation energy of the interface, thereby improving the toughness of the material. In addition, the phase interface in the microphase separation structure can be used as a filler to fill the cracks and cavities of the material, thereby increasing the material’s toughness, density and strength. When the CO/TDI content of the filler continues to increase, the cross-linking degree of the polyurethane network is formed to be greater, and the interfacial adhesion with PBAT is weakened. It can be seen that some holes appear on the quenched surface of the composite PBAT-CO/TDI-30 and protruding particles, the adhesion of the phase interface is weakened, and the existence of holes can be regarded as small defects, which will lead to stress concentration and make the material bear higher stress in the area around the hole. Therefore, the plastic deformation or fracture of the material in these areas risks increases, and the strength of the material is reduced, which is consistent with the law of mechanical properties of the composite.

## 4. Conclusions

In this study, PBAT-CO/TDI composites with different ratios were prepared using melt-blending and hot-pressing technology, and the strengthening and toughening mechanism of the composites was explored. At present, in terms of the modification process and fillers of PBAT, some reports mainly use some expensive nanoparticles to enhance the strength of PBAT. There are also some problems with the modification method of PBAT. The main ones are that some by-products may be produced during the chemical modification process, or the cost of fillers is high. In addition, the modification method is too complicated and requires a lot of waste of manpower and material resources. As for the modification results, the report mainly improves the strength of PBAT, but the disadvantage is that it will lose part of the toughness. We mainly use melt-blending and hot-pressing molding technologies, which are simple and fast to operate, low in cost and significantly improve mechanical properties. The results showed that CO and TDI reacted in situ in the PBAT matrix to form a castor-oil-based polyurethane cross-linked network structure, the tensile strength of the composite material was 86.19% higher than that of pure PBAT, and the elongation at break was 70.09% higher than that of PBAT. The toughening and strengthening mechanism is to control the composition and morphology of the material so that different components present a microphase separation structure on the scale, which can produce synergistic effects on different scales, thereby improving the mechanical properties of the composite material. Finally, by improving the compatibility between different components, a melt-blended bio-based polyester composite material with a simple and easy-to-operate preparation method and high performance can be obtained. The present study has expanded the market and application scope of PBAT to a certain extent.

## Figures and Tables

**Table 1:** Experimental instruments.

Equipment Name	Model	Manufacturer
Hot press	R302	China Wuhan Qien Technology Co.
Dynamic mechanical thermal analyzer	Q800	TA Instruments, Wilmington (Delaware), USA
Differential scanning calorimeter	DSC-214	Selbnech Instruments, Germany
NMR spectrometer	600 MHz	NETZSCH GmbH, Bavaria, Germany.
Universal testing machine	CMT6503	China Shenzhen Sansi Testing Instrument Co.
X-ray diffractometer	D8-ADVANCE	NETZSCH GmbH, Bavaria, Germany.
Fourier transform infrared spectrometer	RF-5301PC	Shimadzu Manufacturing, Kyoto, Japan
Rotational rheometer	TA DHR-1	TA Instruments, Wilmington (Delaware), USA
Internal mixer	SU-7	China Changzhou Suyan Technology Co.
High speed universal pulverizer	FW100	China Tianjin Tester Co.
Thermogravimetric analyzer	Q500	TA Instruments, Wilmington (Delaware), USA
Scanning electron microscope	S4800	Hitachi, Tokyo, Japan

**Table 2:** DSC data of PBAT and complex PBAT-CO/TDI.

Samples	T_g_ (°C)	T_c_ (°C)	T_m_ (°C)	ΔH_m_ (J/g)	χ_c_DSC (%)
PBAT	−36.4	79.1	121.5	8.59	7.53
CO/TDI-5	−30.4	74.5	121.1	13.38	12.35
CO/TDI-10	−28.7	62.9	118.1	13.87	13.51
CO/TDI-15	−26.0	40.7	112.9	14.11	14.56
CO/TDI-20	−26.4	44.2	112.8	13.22	14.49
CO/TDI-25	−26.9	48.7	112.9	12.23	14.30
CO/TDI-30	−26.1	48.8	113.2	11.33	14.19

**Table 3:** TG data of PBAT and PBAT-CO/TDI complex.

Sample	T_5%_ (°C)	T_max_ (°C)	Residual Mass (600 °C) (%)
PBAT	351.3	399.7	5.81
PBAT-CO/TDI-5	342.83	401.33	5.42
PBAT-CO/TDI-10	336.66	401	8.75
PBAT-CO/TDI-15	318.66	399.5	5.70
PBAT-CO/TDI-20	307	399.83	5.38
PBAT-CO/TDI-25	302.83	399.5	4.32
PBAT-CO/TDI-30	298.66	400.66	3.96

**Table 4:** DMA data of PBAT and complex PBAT-CO/TDI.

Sample	E′	E″ (MPa)	Tg ^a^ (°C)	Tg ^b^ (°C)
PBAT	2438.57	81.60	−26.25	−22.04
PBAT−CO/TDI−5	2494.27	70.87	−23.95	−19.62
PBAT−CO/TDI−10	2455.45	73.00	−23.74	−17.39
PBAT−CO/TDI−15	2416.03	68.66	−20.85	−14.22
PBAT−CO/TDI−20	2359.46	67.47	−21.11	−15.11
PBAT−CO/TDI−25	2272.06	72.86	−21.22	−15.94
PBAT−CO/TDI−30	2361.76	73.71	−20.71	−15.59

Tg ^a^: Glass transition temperature in loss modulus curves; Tg ^b^: Glass transition temperature in the loss factor curve.

## Data Availability

The data that support the findings of this study are available from the corresponding author upon reasonable request.
